# Tunable Drug Release from Fused Deposition Modelling (FDM) 3D-Printed Tablets Fabricated Using a Novel Extrudable Polymer

**DOI:** 10.3390/pharmaceutics14102192

**Published:** 2022-10-14

**Authors:** Vishvesh Raje, Siddhant Palekar, Sabrina Banella, Ketan Patel

**Affiliations:** College of Pharmacy and Health Sciences, St. John’s University, Queens, NY 11439, USA

**Keywords:** poly(2-ethyl-2-oxazoline), FDM 3D printing, tunable drug release, shell numbers

## Abstract

Three-dimensional (3D) printing is proving to be a pivotal technology for developing personalized dosage forms with bench to bedside feasibility. Fused deposition modelling (FDM) 3D printing has emerged as the most used technique wherein molten drug-loaded polymer filaments are deposited layer-by-layer to fabricate a predefined shape and internal geometry. However, for precise FDM 3D printing, it is imperative for the filaments to have peculiar mechanical/physicochemical properties, which the majority of the FDA/GRAS approved polymers lack. In the current study, a novel water-soluble polymer, Poly(2-ethyl-tetra-oxazoline) [PETOx] has been investigated as an extrudable and printable polymer with two different types of drug molecule—dextromethorphan hydrobromide (DXM) and hydrochlorothiazide (HCTZ). Hot-stage microscopy experiments of drug:polymer (1:1 *w*/*w*) and filaments were carried out at 25–275 °C. HCTZ-loaded filament showed higher toughness of 17 ± 3.25 × 10^6^ J/m^3^ compared with DXM and drug-free filament. Moisture sorption and flexural analysis was performed to understand the correlation of mechanical properties and storage humidity to printability. Varying the number of outer perimeters of each layer (shell number) was observed to affect the drug release pattern from the printlets. The DXM one-shell printlet showed >80%, whereas the DXM five-shell printlet showed >60% of the drug release within 60 min. PETOx could prove to be a high-performance and versatile 3D printable polymer.

## 1. Introduction

Newer and more efficient patient-centric technologies such as multi-particulate formulations, fixed-dose combinations, and 3D printing have been the need of the hour during the last decade [1]. To develop such novel technologies, different drug delivery systems have emerged. For many years now, the oral route of delivery has been the most commonly used, prescribed, and preferred route of delivery. This, in turn, puts tablet dosage forms in the spotlight. However, conventional tablet dosage forms lack the feasibility to develop patient-centric/specific dosage forms; hence, additive manufacturing technologies such as 3D printing have gained attention. For the past few years, 3D printing in medicine has surfaced and has proven to unfold new avenues for personalized/individualized therapy. In addition to customization of dosage, 3D-printed formulations can be exploited/fabricated in a way such that the formulator has total control over the drug release kinetics, size of the formulation, shape of the formulation, accurate dosing and most importantly developing formulations that are ‘individualized’. For many years, traditional therapy has focused on only one thing, ‘one size fits all’, however, the fact of the matter is that every individual/patient is unique, and ‘one size does not fit all’. Hence, it is imperative to dwell and focus the attention on formulation research and development in additive manufacturing technologies to improve overall patient therapy. To classify the field of 3D printing, there are different printers based on the application and desired result. For, e.g., fused deposition modeling (FDM), stereolithography (SLA), selective laser sintering (SLS), inkjet, drop-on-solid (DOS)/Spritam technology, etc. Among all of these, due to its inherent inexpensiveness and rapid prototyping, FDM 3D printing has gained a lot of interest from pharmaceutical academic and industrial research and development. Recently, Triastek industries developed a MEDTM (melt extrusion deposition) 3D printing technology that can construct tablets with various shapes as well as internal geometric structures [2].

FDM 3D printing technology is based on the nozzle-based deposition of molten filament in a layer-by-layer manner. It provides control over the engineering of the structure to be printed. This allows the formulator freedom to work with the personalization of the formulation based on the weight, shape, infill percentage, etc. Pharmaceutical application of FDM requires drug-incorporated polymeric filaments. To fabricate these filaments, melt extrusion is an imperative step. Hot-melt extrusion (HME) technology has been used in the polymer and food manufacturing industry for many years, wherein the raw material is processed at elevated temperatures and uniformly mixed [3]. To obtain drug-loaded filaments, a mixture of drug and polymer is fed in the hopper of the heated barrel equipped with a unique screw geometry. The screw design is configured such that there is adequate mixing and possible amorphization of the drug in the polymeric matrix. Different polymers such as polylactic acid (PLA), polyvinyl alcohol (PVA), polyvinyl alcohol–polyethylene glycol graft (Kollicoat^®^IR), hydroxypropyl cellulose (HPC), hydroxypropyl methylcellulose (HPMC), ethyl cellulose (EC), hydroxypropyl methylcellulose acetate succinate (HPMCAS), polyvinyl caprolactam-polyvinyl acetate-polyethylene glycol graft copolymer (Soluplus^®^), etc., have been researched as carriers or matrices for drugs in 3D printing [4,5,6,7]. For successful 3D printing, filaments with optimum thermal stability are essential. As well as to ensure passage through the printer nozzle and gears appropriate mechanical properties such as flexibility, strength, and melt viscosity are required. Most pharmaceutical-grade polymers are either brittle which breaks in the gear of the printer or delicate which cannot be pushed by the gear making these polymers not printable using FDM [8]. As an alternative to making these polymers, printable other additives or plasticizers are used [9,10]. The use of additives or plasticizers may lead to a complex formulation and increase the chances of adverse stability of the formulation. Adding more to the story, major 3D-printed tablets display slow and mostly incomplete drug releases. To tackle the problems associated with 3D printing and release, the pharmaceutical field is always in search of new polymers with ideal chemistry and material properties. One such family of polymers is poly (2-alkyl-oxazolines). 

Recently, the poly(2-alkyl-2-oxazoline) (PAOx) polymer class has emerged to be a choice carrier in drug delivery due to its versatility. PAOx-based material has been extensively explored for protein-drug conjugates, nanoparticles, hydrogels, polymersomes, micelles, and hot-melt extrusion. PAOxs can be synthesized by cationic ring opening polymerization of 2-substituted 4,5-dihydroxyoxazoles, called 2-oxazolines. PAOxs have remarkably high physical-chemical stability and are also biocompatible. The PAOx with ethyl side chain namely poly(2-ethyl-2-oxazoline) (PEtOx) has already been explored as a versatile polymer for different formulation strategies. Previous reports exploring the application of PETOx have involved the fabrication of amorphous solid dispersions for fenofibrate and itraconazole [11]. Apart from that, PETOx has been also reported to help in controlling the rate of release of hydrophilic drugs such as metoprolol tartrate after injection molding [12]. A recent report by Feng et al. aimed to develop 3D-printed tablets of acetaminophen with a mixture of PETOx and Eudragit^®^RL [13]. The current study aims to study and explore the feasibility of PETOx being extruded and printed without any additive inert excipients. Apart from this, our research focuses on the effect of the number of shells on drug release patterns from 3D-printed tablets. This research article is one of the first reports on the printing of PETOx by itself and to study the effect of shell numbers. For the same purpose, drug-loaded filaments of one low melting-highly water-soluble drug, dextromethorphan hydrobromide (DXM), and a high melting-poorly water-soluble drug, hydrochlorothiazide (HCTZ) were formulated with PETOx, respectively and characterized for their printability and release. DXM and HCTZ were chosen as model drugs due to their vastly different physicochemical properties such as solubility, melting point, logP, etc. The other important rationale for such different drugs to be selected was to assess the performance of the polymer with different moieties. 

There have been reports of modulating drug release using tablet wall [14,15], but to change the tablet’s wall thickness it is necessary to design a whole new tablet with desired dimensions of internal-external diameter, top, and bottom thickness. Instead, using shell numbers, an easy assessment of adjusting wall thickness can be done. Each shell has a default thickness and changing the number of shells can modulate the thickness of the wall of the printlet. This follows a change in the drug release profile of the printlets. A complete drug release was obtained using PETOx as a carrier polymer and without the use of other excipients or plasticizers. The current study explores the potential application of PETOx for formulating immediate-release formulations using FDM 3D printing technology.

## 2. Materials and Methods

The Dextromethorphan HBr (>98%) (DXM) was acquired from AK Scientific (Union City, CA, USA), and Hydrochlorothiazide (>97%) (HCTZ) was acquired from Tokyo Chemical Industry (St. Portland, OR, USA). Poly(2-ethyl-2-oxazoline) (Aquazol^®^) was kindly gifted by Polymer Chemistry Innovation Inc. (Tucson, AZ, USA). HME Cleaner Plus was used to clean the extruder after experiments were gifted by BioGrund GmBh. Acetonitrile (HPLC grade), Hydrochloric Acid (HCl) were acquired from Fisher Scientific (Hampton, NH, USA).

### 2.1. UV Analysis

Development of a standard curve for DXM and HCTZ was done using a UV spectrophotometer (DS-C, DeNovix Inc., Wilmington, DE, USA). For DXM, accurately weighed (10 mg) DXM was dissolved in 10 mL of distilled water to prepare a stock solution of 1 mg/mL. For HCTZ, 10 mg was dissolved in a 10 mL solution of acetonitrile: water (50:50, *v*/*v*) to prepare a stock solution of 1 mg/mL. These stock solutions were serially diluted appropriately. λ_max_ of 278 nm for DXM [16] and 272 nm for HCTZ were used to analyze the solutions [17]. Triplicates were used for all the sample analysis.

For the assay, a printlet equivalent to 30 mg of the drug was accurately weighed in a 50 mL volumetric flask. 25 mL of 0.1 N hydrochloric acid (HCl) was added and then samples were sonicated for 15 min. The achieved clear solution was diluted with the respective solvents (H_2_O for DXM and ACN: H_2_O-50:50 *v*/*v* for HCTZ) to make up the volume (50 mL). The solutions were centrifuged, and appropriately diluted and the absorbance was analyzed at determined λ_max_. The resultant %drug was calculated using the generated calibration curve [17].

### 2.2. Hot-Stage Microscopy—Polymer and Drug

DXM/HCTZ, PETOX, DXM–PETOX (1:1 *w*/*w*), and HCTZ–PETOX (1:1 *w*/*w*) powders were loaded on the glass slide and placed on the hot-stage assembly (Mettler-Toledo LLC, Columbus, OH, USA). A ratio of 1:1 *w*/*w* was selected to maximize the probability of observing interaction and changes [18]. The samples were heated at the rate of 10 °C/min, from 30 °C to 300 °C, and observed at 10× magnification under a polarized light microscope (Eclipse^®^ 50i, Nikon Inc., Tokyo, Japan) for any melting of solid particles and the possible loss of birefringence of any crystalline material present as a function of temperature [19].

### 2.3. Hot-Melt Extrusion

Drug-loaded filaments were prepared using hot-melt extrusion. However, to achieve a uniform particle size, the obtained polymer was milled using an analytical mill (Tekmar Company, Mason, OH, USA). The powder was passed through the #35 size mesh for uniform particle size and then used for further studies. An 11 mm parallel twin screw extruder (Thermo Scientific, Waltham, MA, USA) was used to perform melt extrusion of DXM–PETOx (1:9) and HCTZ–PETOx (1:9). Primarily, powder blends were prepared by accurately weighing and mixing in a Turbula^®^mixer (Willy A. Bachofen, Switzerland). After mixing, the melt extrusion was performed using a medium shear screw design. The screw design was configured to have two-kneading zones, one high-shear zone with an offset angle of 90° and one low-shear zone with an offset angle of 60°. For hot melt extrusion the powder was fed at a feed rate of 2 g/min to the heated barrel. Heating zones were set with increasing temperatures from 80 °C to 170 °C (R → L) for DXM and from 130 °C to 200 °C (R → L). FDM 3D printer required specific diameter of <2 mm for filaments. Hence, an extrusion die having an internal diameter of 1.55 mm was selected for HME. Screw speed of 100 rpm was used for extrusion [17,20,21,22,23,24].

### 2.4. Three-Point Bend Test

Mechanical properties of drug-loaded filaments were characterized using three-point bend test. The study was performed according to the previous report as Palekar et al. [22]. A TA.XTplus Texture Analyzer (Texture Technologies Corp., Stable Micro Systems Ltd., South Hamilton, MA, USA) was used for the analysis. The mechanical properties of drug-loaded filaments were compared against polylactic acid (PLA) which is a commercially used polymer for FDM 3D printing. 

### 2.5. Solid-State Characterization

Solid state characterization of neat drugs, polymer, physical mixture, and formulated extrudates were performed. 

#### 2.5.1. Differential Scanning Calorimetry (DSC)

A standard differential scanning calorimeter (DSC Q200 TA instruments, Newcastle, DE, USA) was used for thermal assessments. Samples were weighed accurately (5 mg) and hermetically sealed in TA aluminum pans. The procedure involved equilibration of sample at 25 °C for 5 min and then using a ramp of 10 °C/min sample was heated up to temperature of 300 °C. The analysis of the data was done using TA 200 analysis software [25].

#### 2.5.2. X-ray Powder Diffraction (XRPD)

A Shimadzu 6000 X-ray Diffractor (Shimadzu Corporation, Kyoto, Japan) was used for XRPD. For scanning range of 10–40° (2θ) a CuKa, monochromatic radiation source emitting X-ray radiation at 60 kV and 55 mA was used. Glass sample holders with cavities were used to place the samples. To obtain smooth and uniform surfaces samples were compressed [20]. 

### 2.6. Design and 3D Printing

The design of 3D tablet (printlet) was prepared using TinkerCad online designing software and exported as a stereolithographic (.stl) file. This file was used in the MakerBot print software for printing parameters modification. The outer nozzle diameter of 0.4 mm was set to print the printlet. The printing temperature of 200 °C was calibrated for the best printing results. Printlet size was set to be [X:Y:Z] (10:10:7 mm). The number of outer perimeters of each layer briefly called “shell number” was varied to obtain two types of printlets. Printlets with shell numbers 1 and 5 were printed. All other parameters such as build plate temperature, floor, roof solid and surface thickness, filament cooling fan speed, layer height, infill density, and infill pattern were optimized to the values mentioned in Table 1. For HCTZ, one of the groups with 1 shell, was chosen to have an infill density of 95% and tablet size as [X:Y:Z] (8:8:5.6 mm). These compact 1-shell printlets had a similar weight to the original 1-shell printlet of HCTZ with size [X:Y:Z] (10:10:7 mm). All printlets were stored in sealed glass vials.

### 2.7. Moisture Sorption Analysis

To evaluate the moisture sorption, dynamic vapor sorption analysis was performed using VTI-SA^+^ (TA Instruments, Wilmington, DE, USA). The moisture sorption was observed at 25 °C of drugs, PETOX, PLA as a control and extruded filaments. For analysis, each sample was placed on a quartz pan (25 ± 5 mg) and dried at 40 °C for 60 min under nitrogen flow, to reach 0% relative humidity (RH%). Subsequently, RH was automatically increased up to 100% in steps of 10% RH, when equilibrium (0.1% per 5 min) was reached for the set climate stage. A maximum time of 20 min was given to reach the threshold [26].

### 2.8. In Vitro Dissolution

A 250 mL of 0.1N HCl (pH 1.2) maintained at a temperature of 37 ± 0.5 °C was used for dissolution media to access the in vitro, drug release from the printlets. A bathless USP II dissolution apparatus (Symphony 7100, Distek, New Brunswick, NJ, USA) was used for all experiments. A paddle speed of 50 rpm were used for constant stirring and dissolution medium and. The same experimental conditions were used for all the different printlets. Samples (4 mL) were withdrawn at predetermined time intervals of 15, 30, 45, 60, 90, and 120 min. At each time point, an equal volume of fresh dissolution medium (maintained at 37 °C) was replaced in the dissolution vessels. Previously developed standard curve was used to analyze the %drug release [27,28,29].

## 3. Results and Discussion

### 3.1. Hot-Stage Microscopy of Drugs and Drug-PETOx Physical Mixture

**Figure 1:**
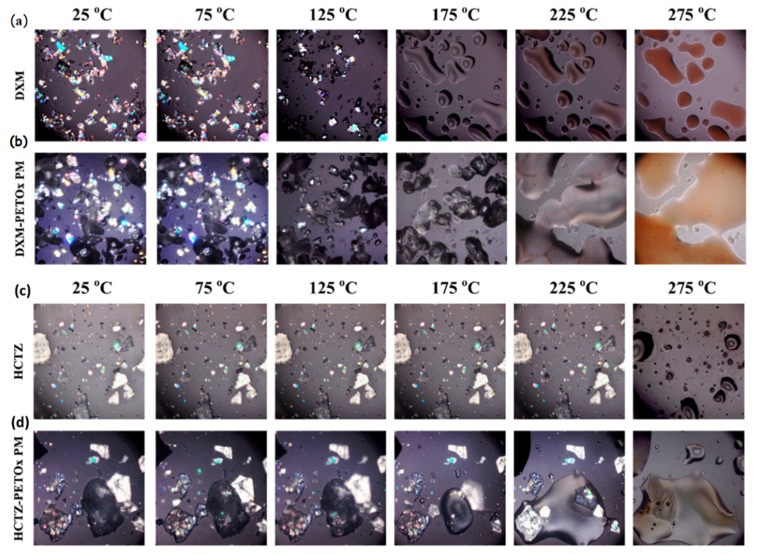
Hot-stage microscopy of powdered samples of (**a**) DXM, (**b**) DXM with PETOx, (**c**) HCTZ, (**d**) HCTZ with PETOx.

Melting is one of the major elements of FDM 3D printing as well as HME. Henceforth, it is essential to estimate the drug’s characterization at processing temperatures. Hot-stage microscopy is one of the techniques to monitor the melting process of samples visually. Figure 1a,b show the hot-stage microscopic images of DXM and 1:1 *w*/*w* physical mixture of DXM and PETOx, whereas Figure 1c,d show images of 1:1 *w*/*w* physical mixture of HCTZ and PETOx at various temperatures. It was noticed, that at the exact melting point of drugs, i.e., at 125 °C and 275 °C DXM and HCTZ crystals converted into the liquid state, respectively. PETOx being a translucent-semicrystalline polymer, evident melting was not observed at around 60 °C-glass transition temperature (T_g_) of PETOx. The clear transition of the PETOx powder particles into a rubbery state was witnessed at 175 °C in both the samples, DXM–PETOx and HCTZ–PETOx physical mixtures. At 275 °C, a reddish-brownish color was observed in the case of DXM and DXM–PETOx physical mixture. 

The microscopic images of discernible melting of DXM and HCTZ exactly at the melting point justify the DSC endotherms at the same temperature. However, in the case of PETOx evident melting was observed at 175 °C. The appearance of solid particles of HCTZ crystals and semisolid rubbery PETOx at 175 °C can be responsible for the higher torque during the extrusion at temperatures lower than 175 °C. High viscosity and low melting of the polymer can be the reason for higher torque for HME [30]. Hence, for the HME of HCTZ–PETOx, a higher temperature range than DXM–PETOx might be required to reduce the torque. As it can also be noted in the hot-stage microscopy that DXM crystals melted and disappeared before 175 °C. In a way, it is beneficial to have melted drugs for molecular dispersion of polymer and drugs during HME [31].

### 3.2. Hot Melt Extrusion

From preliminary study of DSC, T_g_ of PETOx was observed to be 60 °C. It is usually assumed that the extrusion temperature for a polymer should be 20–30 °C more than the T_g_ of the polymer [32,33]. Based on this rule, extrusion temperatures were selected. DXM has a low melting point at 125 °C compared with HCTZ at 275 °C; hence, the extrusion of DXM–PETOx required lower temperatures compared with HCTZ–PETOx. Primarily, similar extrusion temperatures (190 °C) were used. For temperatures higher than 180 °C, DXM–PETOx porous-brittle air entrapped filaments were obtained. Hence, a lower 170 °C was selected as an ideal temperature for the extrusion of DXM–PETOx. The torque during the extrusion was 18–20%. In the case of HCTZ–PETOx, at a temperature of 190 °C, a higher torque of 95–99% was observed may be due to the improper melting of the material. To circumvent the same, the temperature was increased to 200 °C. At this temperature, the %torque relatively decreased to 70–74%. Clear glassy elastic filaments were achieved at this temperature. 

Boel et al. reported the successful hot-melt extrusion of PETOx with the drugs fenofibrate (FFB) and itraconazole (ITC) forming an amorphous solid dispersion [11]. The melting point of the drugs FFB and ITC affected the extrusion temperatures. The barrel temperature was set to be 170 °C for ITC (above its melting point: 168 °C), whereas for FFB (melting point: 80 °C) temperature of the barrel used was 85 °C. This aligns with the observations of the current study. At higher temperatures, a transition of PETOx into molten form was prominently seen in hot-stage microscopy. At temperature 275 °C, the reddish-brownish color was observed in the case of DXM as well as the DEX-PETOx physical mixture. Similar colored and air entrapped extrudates were obtained after the HME of DXM–PETOx. The hot-stage microscopy supports the assumption of overheating of the mixture during the HME which led to instability of the physical mixture and failure of extrusion [34]. DXM has a low melting point at 125 °C. Moreover, as seen in the hot-stage microscopy, at a temperature of 175 °C, the drug has melted completely, whereas PETOx is in the rubbery state/softened/not completely melted. Melting of the drug at a lower temperature results plasticization effect [31]. The plasticization effect results in lower torque during the HME [35]. The lower torque of 18–20% during the HME process of DXM–PETOx at temperatures below 170 °C, may be attributed to the result of the plasticization effect of DXM. In the other scenario, HCTZ has a higher melting point of 275 °C. Usually, to obtain a molecular dispersion, the extrusion temperature should be greater than the melting point of the drug [36]. However, if the drug has affirmative interactions such as hydrogen bonding, the polymer can dissolve the drug lower than its melting point [37]. DSC thermogram of HCTZ with PETOx physical mixture shows significant depression in the melting point of HCTZ. These can be an indication of favorable HCTZ–PETOx interaction. As a result, clear glassy filaments were obtained after a successful HME of HCTZ with PETOx at a temperature of 200 °C.

### 3.3. Hot-Stage Microscopy of Extrudates

**Figure 2:**
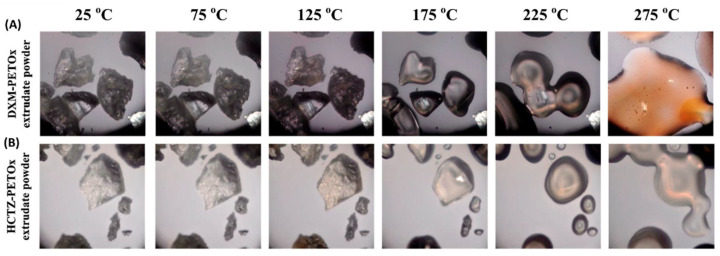
Hot-stage microscopy of powdered samples of (**A**) DXM extrudates and (**B**) HCTZ extrudates.

Figure 2A,B present hot-stage microscopic images of DXM and HCTZ powdered extrudes. The transition of extrudates from solid state to rubbery/liquid state was witnessed at 175 °C and a complete liquid state was observed at 225 °C.

FDM 3D printer did not have the similar assembly of rotating twin screw of HME, and hence, the same processing temperatures cannot be used for the printing. While determining the printing temperature of the drug-loaded filament, it was essential to check the melting temperature of the filament. Drug-loaded filaments showed rubbery state transition at 175 °C and melting at 225 °C. Hence, a temperature more than or close to 175 °C can be a possible printing temperature for the drug-loaded filaments. 

### 3.4. Three-Point Bend Test

**Figure 3:**
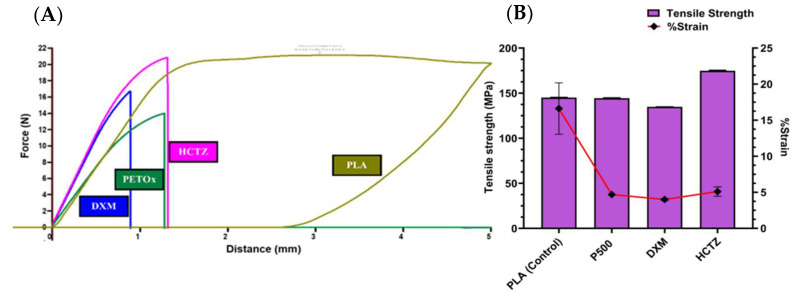
Mechanical characterization of extrudates (**A**) Raw data of constant distribution of force against distance for filaments; DXM–PETOx, HCTZ–PETOx, PETOx, PLA. (**B**) Stress–strain relationship of the filaments.

Mechanical characterizations using a three-point bend test for the extruded filaments are shown in Figure 3A,B. The filaments had a tensile strength between 134.8 ± 0.1 and 174.8 ± 0.6 MPa with a corresponding %strain between 4 ± 0.1 and 5.1 ± 0.6%. Other mechanical properties of filaments such as F_max_, D_max_, Young’s modulus, and toughness, exhibited a similar difference. HCTZ-loaded filaments have a higher toughness of 17 ± 3.25 × 10^6^ J/m^3^ than two other filaments viz. DXM-loaded filaments and neat PETOx filaments (Table 2). However, in the case of DXM-loaded filaments, the toughness was the lowest suggesting that a drug property can change the mechanical properties of filament in turn. The toughness of all the filaments cannot be compared with PLA, which is common in case of pharmaceutical-grade polymers [38]. D_max_ for the filaments was significantly different than the PLA. PLA showed breaking at the distance of 3.52 ± 0.75 mm, whereas neat PETOx filament showed 1.144 ± 0.12 mm. Similar significant differences between PLA and drug-loaded filaments were observed for the D_max_. However, there was no significant difference between the tensile strength of the PLA filaments, neat PETOx filaments, and DXM-loaded filaments (*p* > 0.05). Figure 3A represents the raw data of the constant distribution of force against the entire distance. 

Appropriate mechanical properties of the filaments have a prominent role in successful printing. The gears of the 3D printer serve the purpose of feeding the printing extruder with the required rate of filament material. A filament should have optimum strength and elasticity to pass through the gears of the FDM 3D printer. Polylactic acid (PLA) which is a commercially available non—pharmaceutical filament, is widely used for FDM 3D printing. Hence, the mechanical properties of extruded filaments were compared with PLA. A use of three-point bend test for analyzing the mechanical properties of the filament has reported previously by Palekar et al., Zhang et al. [8]. The pressure applied by the rig of the texture analyzer mimics the pressure applied by the gears of the printer. The material will be under tension or stretched while moving through the printing gears; hence, tensile strength is one of the important parameters in determining the printability of a polymer. The mechanical properties of the filaments were analyzed using tensile strength by Goyanes et al. [39]. The tensile strength of the drug-loaded filaments more than or similar to PLA shows the ability of filaments to withstand the pressure applied by the gears of the printer, leading to successful 3D printing. In addition, neat PETOx, DXM-loaded and HCTZ-loaded filaments have comparable F_max_ with the PLA filaments. However, there was a significant difference between the D_max_ of the filaments and PLA. This breaking distance was observed at the maximum force. As during the 3D printing of filaments there was no breaking observed, it can be considered that the force applied by the gears of printer or overall printing assembly was less than the F_max_. However, filaments might be susceptible to breaking during the storage and handling.

The Young’s modulus is one of the important parameters which can predict the stiffness of the filament. The Young’s modulus of PLA is similar to neat PETOx filament, whereas an increase in the young’s modulus was observed for drug-loaded filaments of DXM and HCTZ. The characteristics of a drug and % *w*/*w* of drug loading have been reported to influence the printability of the filament [40,41]. Report Feng et al. show the inadequacy of PETOx alone as a printable polymer [13]. PETOX combined with acetaminophen at a 20% *w*/*w* loading with PETOX was used. However, in the current study, PETOx with 10% *w/w* loading of DXM and HCTZ separately showed adequate mechanical properties for FDM 3D printing. It can be concluded that drug loading has a major impact on the mechanical properties of extrudates.

### 3.5. Solid-State Characterization

**Figure 4:**
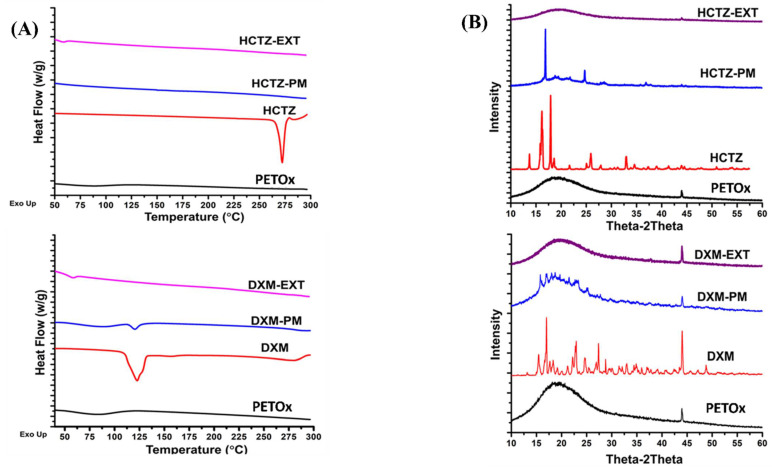
Solid State Characterization of the drug, drug-PETOx physical mixtures, and crushed drug-loaded extrudates by: (**A**) Differential Scanning Calorimetry; (**B**) X-ray powder diffraction.

Figure 4A shows DSC thermograms of the neat drugs, polymer, physical mixture, and formulated extrudates. Pure drugs, DXM, and HCTZ exhibited sharp melting endotherms at 125 °C and 275 °C, respectively. At ~60 °C, a broad endotherm was observed for PETOX confirming T_g_ of the PETOx. In the case of a physical mixture of DXM–PETOx, a less dominant endothermic peak characteristic of DXM can be observed at 125 °C, whereas the HCTZ–PETOx physical mixture showed a significantly depressed melting point at 275 °C, suggesting that HCTZ is miscible with PETOx at all concentrations. The appearance of PETOx endotherm at 60 °C in HME extrudate is in accordance with Van Kuringen et al. [42]. Characteristic endotherms of DXM and HCTZ were absent in crushed extrudates of DXM–PETOx and HCTZ–PETOx. Hence, it was confirmed that the drugs were present in a molecularly dispersed/amorphized state in extrudates. 

XRPD observations complied with DSC results. A broad halo peak at 2θ of 43.98° validated the semi-crystalline nature of PETOx. DXM showed characteristic peaks at 2θ of 21.7°, 22.9°, and 27.36°, whereas HCTZ at 2θ of 16.2°, 18.7°, 27.62°, and 33.78. Similar characteristic halos were observed in the physical mixture. XRPD pattern for DXM and HCTZ crushed extrudates showed a broad halo with the characteristic halos for DXM and HCTZ being absent. Hence, corroborating and confirming that both DXM and HCTZ were present in a molecularly dispersed/amorphized state in the extrudates. 

Hot melt extrusion is extensively explored for the preparation of molecular dispersion of drugs within the polymer [43]. The formation of molecular dispersion of drugs with polymer improves the bioavailability of drug substances [44,45]. As HCTZ has low solubility and low permeability [46], molecular dispersion with a water-soluble polymer such as PETOx can be helpful to improve its solubility. A significantly depressed melting point at 275 °C for HCTZ–PETOx physical mixture, suggests HCTZ is miscible with PETOx [37]. Moreover, the absence of the characteristic endotherm in the DSC, as well as the absence of characteristic halos in XRPD, confirms the molecular dispersion of HCTZ within PETOx. Furthermore, HCTZ and DXM extrudate powder does not show any endotherms which concludes the thermal stability of the filaments at the higher temperature.

### 3.6. Moisture Sorption Analysis

The amount of moisture uptake rate depends on several factors: relative humidity (RH) in the environment, temperature, affinity between the moisture and the surface of the material, and time [47,48]. 3D printing required that the characteristics of extruded filaments remain constant after production and during storage time. Literature has reported that the moisture sorption determines a slight increase in the filament diameter and usually there is a fluctuation in the glass transition temperature (T_g_) [49]. Additionally, for the amorphous material, the uptake of moisture led to a reduction in T_g_ since water acts as a plasticizer agent [50].

APIs, polymer, physical mixture, and hot-melt extruded filaments analyzed show a type III isotherm (according to IUPAC classification). All samples have low moisture uptake at low RH and high moisture sorption at higher RH.

**Figure 5:**
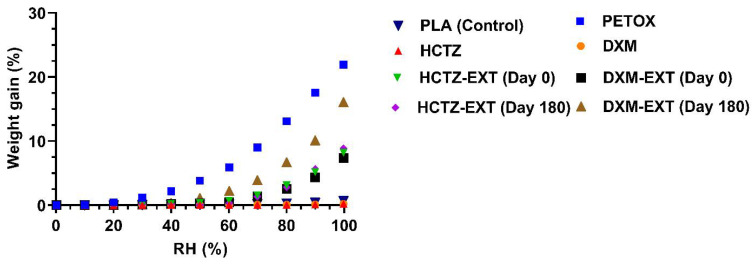
Moisture sorption of the drugs, PETOx, PLA, and crushed drug-loaded extrudate powders at a temperature of 25 °C.

Neat polymer and the physical mixtures showed a higher capacity to uptake moisture than extruded filaments (Figure 5). The different humidity sorption is due to the fact that powders have a greater surface area in comparison with extrudates, and also the porosity is different in powder. All these properties led to a superior moisture uptake from the powders. Thus, the extruded filaments were prepared by processing molten material through the die, a very small orifice, which condensed the material with a reduction in the porosity and surface area. Due to different physical properties, the extrudates have a lower capacity to uptake moisture. Instead, APIs showed a very low uptake of moisture (<0.5% in mass) due to their lipophilic behavior. 

**Figure 6:**
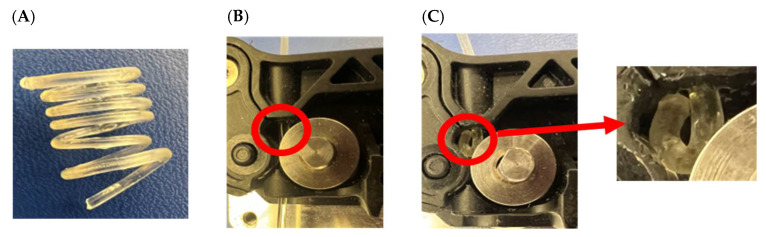
Change in the printability of the DXM extrudate after 180 days of storage. (**A**) Highly elastic filament due to the over-plasticization. (**B**) Filament at day 0 has good printability and easy passage through the gears of the printer. (**C**) Filament on day 180 caused clogging of the gears of the printer and cannot be printed.

Nonetheless, at low RH (≤60% RH) there was <3% moisture sorption by fresh and 6 months-old filaments. However, at greater RH (90 and 100%) the increase in mass (%) was rapidly intensified for all the filaments. For HTCZ filaments, the increases in mass were not significantly different: 5.16% (90% RH) and 8.19% (100% RH) for fresh filaments and 5.67 (90% RH) and 8.88% (RH 100%) for the 6-month-old filaments. Instead, there was a huge difference between DXM extrudates. The fresh one showed a mass increase of 4.36% at 90% RH and 7.38% at 100% RH. In the case of 6 months-old filaments, they reached an increase equal to 10.08% and 16.07% at 90 and 100% RH, respectively. As shown in Figure 6C 6 months-old filaments cannot be printed due to the clogging of the gears. 

However, based on the data obtained from this study, the physical stability of melt extrudates may be protected by shielding them from relatively high humidity. In the actual storage conditions, it appears that filaments tend to uptake moisture turning in rubbery-highly elastic extrudates that are unprintable. Thus, to avoid this issue the extrudate filaments should be stored under 60% RH and 25 °C.

### 3.7. Design and 3D Printing

3D tablets were designed using Tinkercad online designing software. To keep the internal structure uniform and unaffected by any printing parameters, printlets were designed hollow. The dimensions of the printlet were [X:Y:Z] (10:12:5 mm). Other printing parameters were maintained constant except for the shell number for the printlets. The printing parameters used were as mentioned in Table 1. The number of shells was varied for the printlets. Varying the shell number had an impact on the printing time and weight of the printlets. The time required for printing the one-shell printlet was 7 ± 0.2 min while for the five-shell printlets it was 10 ± 0.3 min. The weight of one-shell printlets was 236 ± 6 mg, whereas five-shell printlets weighed 320 ± 5 mg. For the compact HCTZ-loaded printlet, one shell with 95% infill was used. As the dimensions of the compact printlet were smaller [X:Y:Z] (8:8:5.6 mm), the weight was 238 ± 7 mg, which was similar to other one-shell printlets. The printing time of the compact size printlets was 8 ± 0.2 min. The floor and roof surface thickness were kept invariable for all the printlets to assure the constant drug release from the top and bottom of the printlets. 

Initially, following the hot-stage microscopy, a printing temperature of 180 °C was used for the printing of the drug-loaded filaments. Frequent nozzle blocking and failure in printing were obtained. The blocking observed was due to the viscous filament. Hence, the printing temperature was elevated to 190 °C. At this temperature, the problem of clogging was resolved, although significant surface imperfections and displacement of the printlets from the build plate surface were consistent. Calibration of build plate to nozzle distance, increasing build plate temperature till 80 °C to strengthen the adherence of printlet to the bottom surface, did not change the results. Nevertheless, decreasing the printer’s filament cooling fan speed to the lowest at 10% and printing speed to 10 mm/s enabled the printing of better printlets. Decreasing cooling fan speed affirms maintenance of the viscous deposited layer for a longer time due to retention of the higher temperatures on the surface. That gives additional time for a newly formed deposited layer to cling to the previous layer. However, reducing printing speed increases the accuracy of small dimensional edges, and better the deposition of layers. Although this change of parameter assessed better printlets than before, it increased the printing time by more than two-fold the original printing time. Moreover, decreasing cooling fan speed and printing speed had limits and cannot be used further to improve the quality of printlets. Henceforth, the temperature was raised to 200 °C. As desired, the material was flowing continuously, and the fused filaments from the FDM extruder were adhering to each deposited layer uniformly. A perfectly required, surface uniformed printlets were obtained using these printing parameters. Furthermore, 3D-printed tablet showed assay of 98.03 ± 1.64% for DXM, and 98.37 ± 0.83% for HCTZ, which suggest negligible degradation of the drug during the 3D printing.

For manufacturing various tablet-based FDM 3D-printed pharmaceutical dosage forms extensive research has been accomplished. Exploiting different polymer properties to tailor the drug release is a conventional approach and has been explored thoroughly. Various polymers and filaments formulated using HME have been used as feed material for FDM 3D printing [6,21,51,52]. However, FDM demands particular mechanical properties, enforcing added load to adapting formulation to ensure printability. Predominant methods to accomplish desired drug release using FDM 3D printing with the definite/fixed drug concentration in filament entail exploiting tablet shapes, sizes, internal structures, density, etc. The different fabrication patterns which can be enabled via 3D printing have been extensively explored through different reports. The drug release patterns are highly dependent on the fabrication of tablets as mentioned by Nukala et al., wherein tunable release profiles were shown using 60% infill density and various infill patterns viz. hexagonal, diamond for 3D printing of the tablets [17]. Previously Obeid et al. and Reddy Dumpa et al., have reported the use of shell or tablet wall thickness as a parameter to control drug release [14,15]. However, to change the wall thickness of the tablet whole-tablet design needs to be modulated. To change the thickness of the tablet wall, one must consider a change in the parameters such as the inner diameter of the tablet, outer diameter, top and bottom thickness, and infill density. Overall, a newly designed tablet is required each time to change the thickness of the tablet wall. 

**Figure 7:**
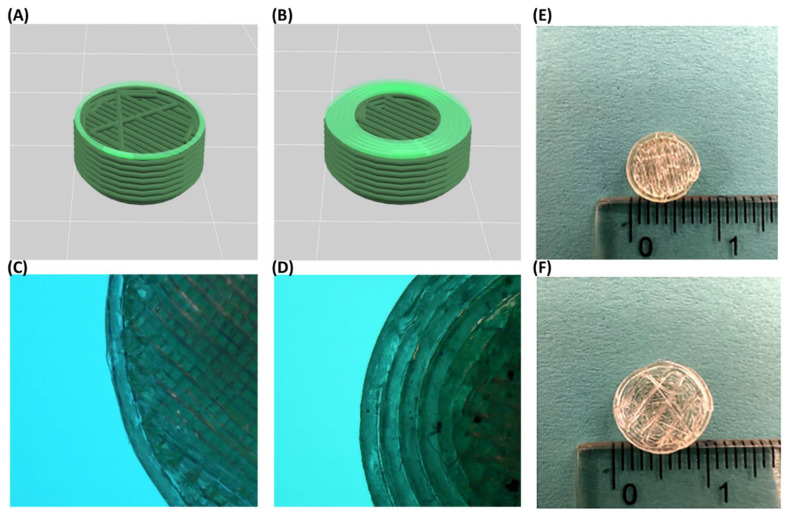
Rendering preview of 3D-printed tablet. (**A**) 1-shell printlet, (**B**) 5-shell printlet. Stereomicroscopic image of section of the (**C**) 1-shell printlet, (**D**) 5-shell printlet. Difference between the size and internal structure of the printlets: (**E**) 1-shell compact printlet with 95% infill (**F**) Printlet with 1 shell and 20% infill.

Shell number is one of the unexplored avenues toward the modulation of drug release using an FDM 3D printer. The number of outer perimeters of each layer briefly called shell number is a printing parameter that has limited/no reports for fabricating a release modulated FDM 3D-printed tablet (Figure 7A,B show rendering preview, whereas Figure 7C,D show stereomicroscopic image of section of one-shell and five-shell printlets, respectively). Using shell numbers, the wall thickness of the tablets can easily be controlled [53]. Each ‘shell’ has a default thickness value (mostly equivalent to the printing nozzle diameter), and the number of these shells can be used to control the thickness of the tablet wall. In the current study, the value used was 0.2 mm which was equivalent to the layer height as well as the inner nozzle diameter of the printer. The number of shells can easily be controlled using printing software. The use of one shell is equivalent to the tablet wall thickness of 0.2 mm, whereas five shells is equivalent to the tablet wall thickness of 1 mm. The additive method 3D printing allows the formulator liberty to formulate the patient-tailored dosage forms. Shell number could be a very promising printing parameter for overcoming design hurdles and modulating release from the same filament. In conclusion, by utilizing a simple drug–polymer matrix, a simple tablet design, and easy change in the single printing parameter that is shell number, tunability of the drug is possible.

### 3.8. In Vitro Dissolution

**Figure 8:**
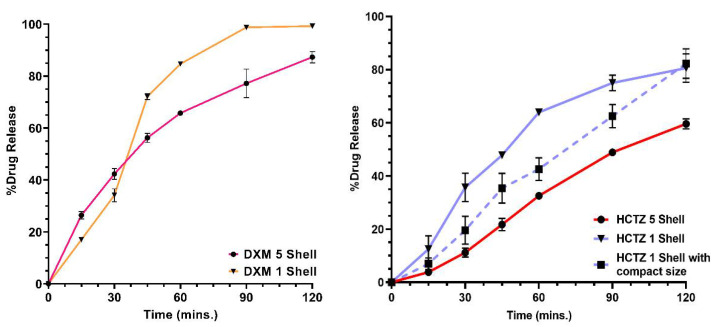
In vitro release of printlets in 250 mL of 0.1 N HCl. The drug release was affected by the drug, infill, size and most importantly the shell number of the printlets.

The dissolution profile of the printlets with different shell numbers and different drugs is shown in Figure 8. A noticeable difference between the drug release of one-shell and five-shell printlets can be seen for both DXM and HCTZ printlets. DXM printlets with 1 shell showed more than 80% of drug release within the first 60 min and total drug release within 90 min. DXM printlets with five shells showed <70% of the drug release within the first 60 min and complete drug release in 150 min. On the other hand, a considerable difference in the drug release of the HCTZ printlets was observed with a change in shell number. HCTZ printlets with one shell showed >60% of the drug release during 60 min and HCTZ printlets with five shells demonstrated a drug release of <40% of the total drug. In the case of the HCTZ compact printlet with one shell, drug release was <50% of the total drug. 

Visual assessment during the dissolution revealed complete disappearance of the outer wall in the case of DXM printlet within 30 min. The printlet was hollow; therefore, it was floating at the start of the dissolution. Once the shells of the printlets were eroded, media entered the internal structure of the printlet and submerged the printlet within the media. PETOx is a highly hygroscopic as well as water-soluble polymer. As soon as the printlet makes contact with media, a viscous layer formation around the printlet can be observed. Furthermore, interaction with media caused erosion of the layer and release of the drug from the polymer matrix. DXM being and hydrophilic drug a quick erosion of printlet was observed compared with HCTZ printlet. HCTZ printlet did not lose integrity the printlet till the first 60 min and 120 min in the case of one-shell and five-shell printlets, respectively. Furthermore, the HCTZ printlet with compact size and 95% infill showed the integrity of the tablet till the end of the dissolution. Figure 7E,F show internal structural differences between compact and normal printlet. The internal structure of the compact printlet had a densely packed polymeric infill, whereas the printlet with 20% infill had a uniform space in-between. The size and infill of the printlet affect the dissolution of the drug [17,22,54]. Thakkar et al. showed higher infill density as a reason for slower drug release from the 3D-printed tablets. Similar, results can be observed in the current study [54]. 

Most of the 3D-printed tablets tend to sink to the bottom of the dissolution vessel and hamper the drug release. Additionally, the most 3D-printed tablet has low tortuosity which is inhibition of water penetration through the formulation. This causes poor or incomplete drug release in most cases. Using water-soluble polymers instead of cellulosic polymers can help to solve the issue. As observed in the current study, even after printlets sank to the bottom, the erosion of the matrix continued, and the drug was released as well. Kempin et al., Palekar et al., and Solanki et al. have demonstrated some of the immediate release formulations [6,22,55]. Even though the compact HCTZ printlet had a dense core with 95% infill, the drug release was complete. Comparing the drug release of the compact printlet with the one-shell 20% infill printlet, it was lower at 60 min, but was higher than the five-shell printlet with 20% infill. Although, HCTZ five-shell printlet had a 20% infill, had a thick wall due to five shells. On the other hand, the one-shell compact printlet had a 95% infill but only one shell. Media can easily penetrate through one shell after a short time, and cause erosion of matrix through the micropores present due to 5% space left between the infill. Therefore, it can be concluded that the number of shells had a greater impact on the drug release profile than the % infill of a printlet.

Feng et al. have also used PETOx as a matrix for the formulation but added other polymers such as Eudragit RLPO, and PEO, in addition to that PEG as a plasticizer [13]. The result of the dissolution study does not align with the current study. This study shows complete drug release within 120–180 min, whereas Feng et al. demonstrate drug release till 24 h [13]. The reason might be the use of other polymers along with the PETOx. Solely, using PETOx with appropriate drug loading can result in printable filaments. Printlets fabricated using such material will result in complete drug release. In addition to this, modulation of the drug release can be performed using easy manipulation of shell numbers. PETOx could be a promising polymer for improving solubility and rapidly dissolving FDM 3D-printed tablets.

## 4. Conclusions

It is difficult to prepare formulations using fused deposition modeling (FDM)-3D printing technology with limited polymer availability. In addition, there is a shortfall of immediate-release polymer materials which have adequate mechanical properties for printing. In the present study, poly(2-ethyl-2-oxazoline) [PETOx] a novel polymer has been exploited for the development of 3D printable filaments. Printing software allowed control over various printing parameters, the number of outer perimeters of each layer (shell number) being one of them. The varying number of shells for the printlets granted control over drug release. PETOx is available with different grades based on the molecular weight of the polymer. Hence, future prospects of study should explore PETOx for physiochemical properties and solubility improvement of poorly water-soluble drug. PETOx can be used for formulating amorphous solid dispersions using HME. Using newer and simpler printing parameters such as shell numbers, and uncomplicated solo polymer materials such as PETOx can enable easygoing steps toward better individualized patient-centric drug formulations.

## Figures and Tables

**Table 1:** Optimized parameters for 3D printing of drug-loaded tablets.

Printing Parameters	Parameter Value
Build plate temperature	50 °C
Floor, roof solid, and surface thickness	1 mm
Filament Cooling Fan Speed	100%
Layer Height	0.2 mm
Number of Shells	1, 5
Infill Density	20%
Infill pattern	Thatch Fill

**Table 2:** Mechanical characteristics of HME extruded filaments.

Batch	F_max_ * (N) ± SD	D_max_ * (mm) ± SD	Young’s Modulus (E) ± SD	Toughness × 10^6^ (J/m^3^) ± SD
PLA	21.074 ± 0.45	3.52 ± 0.75	16.183 ± 0.96	83.9 ± 2.06
PETOx	13.812 ± 0.41	1.144 ± 0.12	14.873 ± 0.65	13.6 ± 1.95
DXM	17.016 ± 0.24	0.889 ± 0.03	21.937 ± 0.54	9.42 ± 0.45
HCTZ	19.389 ± 1.35	1.184 ± 0.14	21.430 ± 1.31	17 ± 3.25

* At maximum force and distance breaking was observed.

## Data Availability

Not applicable.
